# Novel anammox bacteria and nitrogen loss from Lake Superior

**DOI:** 10.1038/s41598-017-12270-1

**Published:** 2017-10-23

**Authors:** Sean A. Crowe, Alexander H. Treusch, Michael Forth, Jiying Li, Cedric Magen, Donald E. Canfield, Bo Thamdrup, Sergei Katsev

**Affiliations:** 1 Departments of Microbiology & Immunology and Earth, Ocean, & Atmospheric Sciences, University of British Columbia, Vancouver, Canada; 2 NordCEE, Department of Biology, University of Southern Denmark, Odense, Denmark; 3 Large Lakes Observatory, University of Minnesota Duluth, Duluth, USA; 4 Chesepeake Biological Laboratory, Center for Environmental Science, University of Maryland, Solomons, USA

## Abstract

Anaerobic ammonium oxidizing (anammox) bacteria own a central position in the global N-cycle, as they have the ability to oxidize NH_4_^+^ to N_2_ under anoxic conditions using NO_2_^−^. They are responsible for up to 50% of all N_2_ released from marine ecosystems into the atmosphere and are thus indispensible for balancing the activity of N-fixing bacteria and completing the marine N-cycle. The contribution, diversity, and impact of anammox bacteria in freshwater ecosystems, however, is largely unknown, confounding assessments of their role in the global N-cycle. Here we report the activity and diversity of anammox bacteria in the world’s largest freshwater lake—Lake Superior. We found that anammox performed by previously undiscovered bacteria is an important contributor to sediment N_2_ production. We observed striking differences in the anammox bacterial populations found at different locations within Lake Superior and those described from other locations. Our data thus reveal that novel anammox bacteria underpin N-loss from Lake Superior, and if more broadly distributed across inland waters would play an important role in continental N-cycling and mitigation of fixed nitrogen transfer from land to the sea.

## Introduction

As one of Earth’s great biogeochemical cycles, the N-cycle intimately couples to the carbon cycle, playing an important role in regulating global primary production^1^. The availability of N often limits productivity in many regions of the ocean, in most soils, and in some large freshwater environments^2,3^. Naturally, N is supplied to these environments through the microbial fixation of atmospheric N_2_ gas into biomass. In aerobic environments, this organic N is oxidized to nitrate, which can be mobilized from soils, and accumulate in groundwater, lakes, rivers, estuaries and the coastal ocean, leading to possible productivity increases and deleterious eutrophication with ensuing hypoxia or anoxia. Ultimate N removal from these environments back to the atmosphere depends on a combination of microbial denitrification and anammox. The anammox reaction is central to fixed nitrogen removal from the oceans where it operates in oxygen minimum zones and sediments playing an outsized role in closing the N-cycle^4^. Its role in the continental N-cycle and its possible activity in freshwater surface environments remains largely unknown.

Anammox bacteria have been detected in freshwater sediments^5,6^ and appear diverse and widely distributed in soils^7–10^. Potential anammox activity, on the other hand, has only been demonstrated in the water column of a single lake, Lake Tanganyika, a stratified, ancient lake situated in East Africa^11^ as well as in eutrophic lake sediments from Japan^6^ and China^8^. As a broader and quantitative test for the distribution and activity of anammox, we have thus combined molecular microbial techniques with geochemical analyses and process rate measurements to chart the diversity and biogeochemical significance of anammox bacteria in the sediments of Lake Superior—the world’s largest lake.

**Figure 1:**
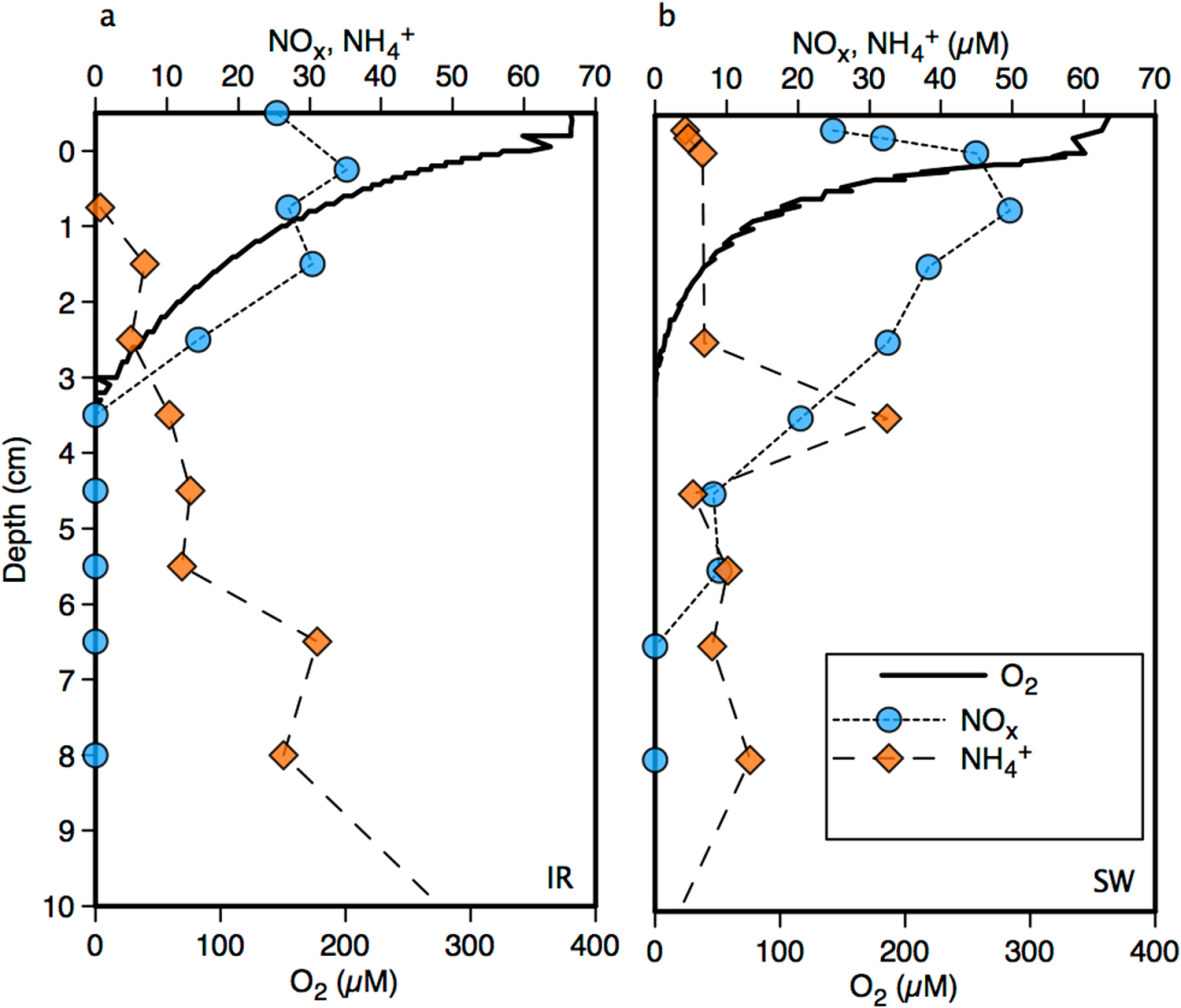
Profiles illustrating the depth distributions of oxygen, NO_x_ (combined nitrate and nitrite), and ammonium at stations IR (**a**) and SW (**b**).

Holding 10% of the world’s surface freshwater, Lake Superior is an important natural resource. It is characterized by low rates of primary production^12^, and its sediments exhibit low rates of biogeochemical activity and very deep oxygen penetration (Fig. 1)^13^. Nitrate concentrations in Lake Superior are high (>20 µmol l^−1^) and have been increasing over the last century^14^. The high nitrate and low phosphate concentrations lead to extreme nutrient stoichiometry and N:P ratios of >10 000^14^. Nitrification in Lake Superior’s sediments^15,16^ leads to accumulation of nitrate (and nitrite) in the upper reaches of the sediment and penetration^16^ of nitrate well into the anoxic deeper sediments where ammonium accumulates (Fig. 1). The juxtaposition of oxidized forms of nitrogen with ammonium leads to an environment favorable to anammox. This pattern holds across multiple locations across the lake, and the nearly constant conditions at these great depths ensure minimal seasonal variability^16^.

We used whole core incubations to measure N_2_ production rates and pathways in Lake Superior’s sediments at two deep-water stations (IR and SW)^17^. The two stations exhibit similar biogeochemical properties, but the deeper sediments at station SW appear to be less active leading to deeper penetration of oxidized nitrogen, and lower ammonium concentrations (Fig. 1). Low total rates of N_2_ production were found at both stations (Table 1) and are consistent with the low overall biogeochemical activity of Lake Superior’s sediments, as indicated by oxygen uptake rates^13^. Higher overall rates of N_2_ production were observed at station IR than at SW, in line with shallower penetration of nitrate (and nitrite) at IR than SW. The anammox reaction was operative at both stations, and indeed dominated N_2_ production at SW, contributing more than 50% of the total N_2_ produced. At station IR, anammox was responsible for 24% of the total N_2_ production. Greater importance of anammox to N_2_ production at station SW compared to IR is in line with the general overall observation that anammox plays a proportionally greater role relative to denitrification in sediments with lower overall biogeochemical activity^18–20^, as for example qualified by oxygen respiration rates. Likewise, absolute rates of anammox at station IR are much higher than at SW despite the fact that its relative contribution to N_2_ production is lower. Similar observations have been made in marine sediments^18^, implying comparable controls on N loss processes in both marine and freshwater environments.

**Table 1:** Measured whole-core rates of N transformations and rates calculated from a N mass balance (mmol m^−2^ d^−1^)^16^.

	Amx.	Denit.	Amx. %	Total N_2_ Meas.	Total N_2_ Calc.
**IR**	0.040	0.128	24	0.17	0.14
**SW**	0.021	0.019	53	0.04	0.06

**Figure 2:**
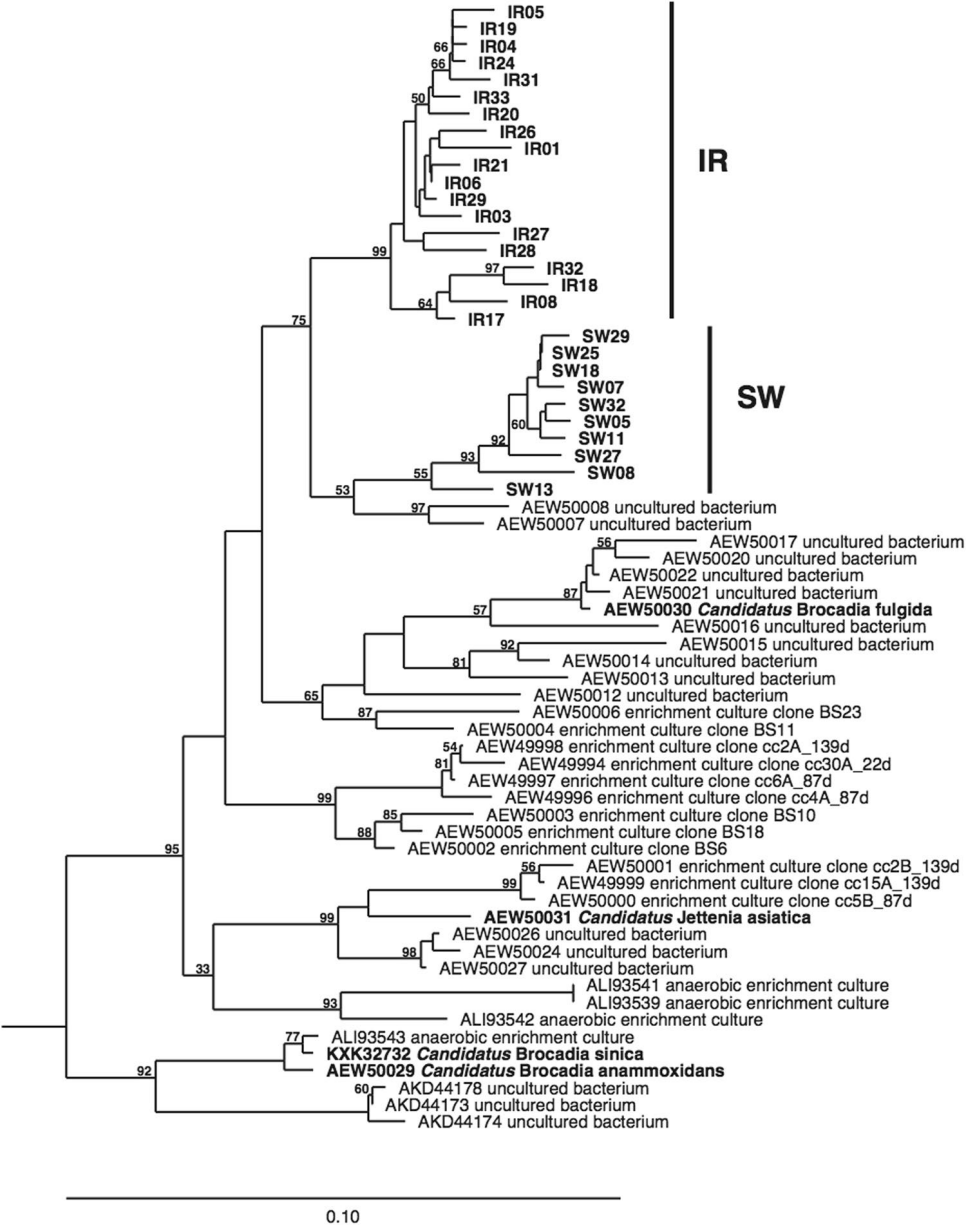
Neighbor joining phylogenetic reconstruction of *Hzs*A sequences from Lake Superior based on 397 positions of an amino acid alignment. Bootstrap values (1000 replicates) above 50% are shown. *Candidatus* Scalindua sp. enrichment culture clone 15L (AEW50032) was used as outgroup.

The difference in the rates and relative importance of anammox observed between sites was also reflected in the diversity of the hydrazine synthase gene (*hzs*A) (Fig. 2), a functional marker for anammox bacteria^21^. From all cultivated anammox bacteria, the *hzs*A from ‘*Candidatus* Brocadia fulgida’ was the closest relative to the *hzs*A sequences we recovered from both stations, which is consistent with the known affinity of *Ca.* Brocadia for non-saline environments. The *hzs*A sequences from the two Lake Superior stations clustered separate from each other, with those from IR forming a novel monophyletic group exclusively containing sequences from this station. The sequences obtained from SW also cluster together, but two sequences from the Ooijpolder eutrophic freshwater ditch sediment^21^ were closely related, indicating a likely wider distribution of these anammox bacteria in freshwater environments. This implies that the diversity of anammox bacteria is underexplored and may be much broader than currently known when freshwater taxa are fully described.

Dissimilatory reduction of nitrate to ammonium (DNRA) can lead to the underestimation of anammox due to the conversion of some ^15^N-NO_3_^−^ to ^15^N-NH_4_^+^, and its incorporation into the^30^N_2_ (^15^N^15^N) pool. We assayed for DNRA in our whole-core incubations, but did not detect it. Our measurements, however, may be blind to DNRA tightly coupled to anammox, such as that observed in sediments underlying marine oxygen minimum zones^22^. We thus take our whole-core measurements as minimum estimates for the contribution of anammox to total N_2_ production.

To verify the anammox activity observed in our whole-core incubations, we measured potential rates of denitrification and anammox in anoxic slurry incubations. In anoxic slurry incubations with added ^15^N-NO_3_^−^, no ^14^N-NO_3_^−^ and in the absence of DNRA, anammox produces only ^29^N_2_ (^14^N^15^N), whereas the only known ^30^N_2_ production pathway is through denitrification. As with our whole core measurements, DNRA was not detected in any of our slurry incubations. Both ^29^N_2_ and ^30^N_2_ were produced in the slurry incubations confirming the activity of anammox in sediments from both stations. As in the whole core incubations, anammox contributed a large fraction (Table 2), up to 57%, to the total N_2_ produced. At IR, the highest rates of N_2_ production, and the highest contribution of anammox were both observed in the upper sediment layer, coinciding with the highest *in situ* nitrate concentrations of all layers. In contrast, the highest rates of N_2_ production, and the highest contribution of anammox were observed in the deeper sediment layer at station SW. This is likely related to a deeper *in situ* nitrate maximum at SW than at IR, which itself is the likely result of lower rates of oxygen consumption and deeper oxygen penetration at SW.

**Table 2:** Potential rates (µmol l^−1^ hr^−1^) of N_2_ production in slurry incubations (1SD in parentheses refers to final decimal place displayed).

	^15^N-NO_3_^−^			^15^N-NH_4_^+^	
	p^29^N_2_	p^30^N_2_	% Amx.	p^29^N_2_	p^30^N_2_
**IR 0–3**	0.4 (1)	0.7 (2)	57	0.012 (1)	0
**IR 3–6**	0.05 (1)	0.3 (2)	21	0	0
**SW 0–3**	0.014 (3)	0.04 (1)	34	0.015 (3)	0
**SW 3–6**	0.04 (2)	0.08 (2)	47	0.008 (3)	0.0002(1)

Anoxic slurry incubations were also conducted with ^15^N-NH_4_^+^ with addition of allylthiourea (ATU) to specifically inhibit of aerobic ammonium oxidation. Only anammox is known to produce ^15^N-labeled N_2_ (^29^N_2_) in incubations with ^15^N-NH_4_^+^. Notably, without added nitrate, anammox in these experiments will be nitrite limited, owing to low rates of nitrite production from the low natural nitrate concentrations present. Again, anammox was observed at both stations, however, it was only observed at the shallowest depth at IR, and then at lower rates than observed in the incubations with added ^15^N-NO_3_^−^. We attribute this to nitrite limitation in the shallow sediment and a complete lack of nitrate and therefore nitrite in the deeper sediment layer at IR. Both SW sediment layers yielded anammox from ^15^N-NH_4_^+^. Natural nitrate concentrations are higher at SW, and indeed, ^15^N-NH_4_^+^ based rates are comparable to ^15^N-NO_3_^−^ based rates for the shallower depth. In contrast, ^15^N-NH_4_^+^ based rates are lower than ^15^N-NO_3_^−^ in the deeper layer, and again, we attribute this to nitrite limitation.

Our experiments also provide information on the anaerobic oxidation of NH_4_^+^ to N_2_ with alternative oxidants, which has been previously proposed^23^, but remains unsubstantiated by a robust set of nitrogen isotope labeling experiments. In our ^15^N-NH_4_^+^ based experiments, the direct oxidation of NH_4_^+^ to N_2_ would be revealed as ^30^N_2_ production, which was absent in all incubations except in the deeper sediment layer from station SW. Here ^30^N_2_ production rates are low, but significant, suggesting low rates of oxidation of NH_4_^+^ to N_2_ under anoxic conditions or nitrification by ATU-insensitive ammonium oxidizing archea. Possible oxidants include the (hydr)oxides of Fe and Mn, which are abundant in station SW sediments^13^. Further work is required to verify this process and the oxidant involved, but in Lake Superior, the rates appear to be very low and insignificant to N-cycling.

**Figure 3:**
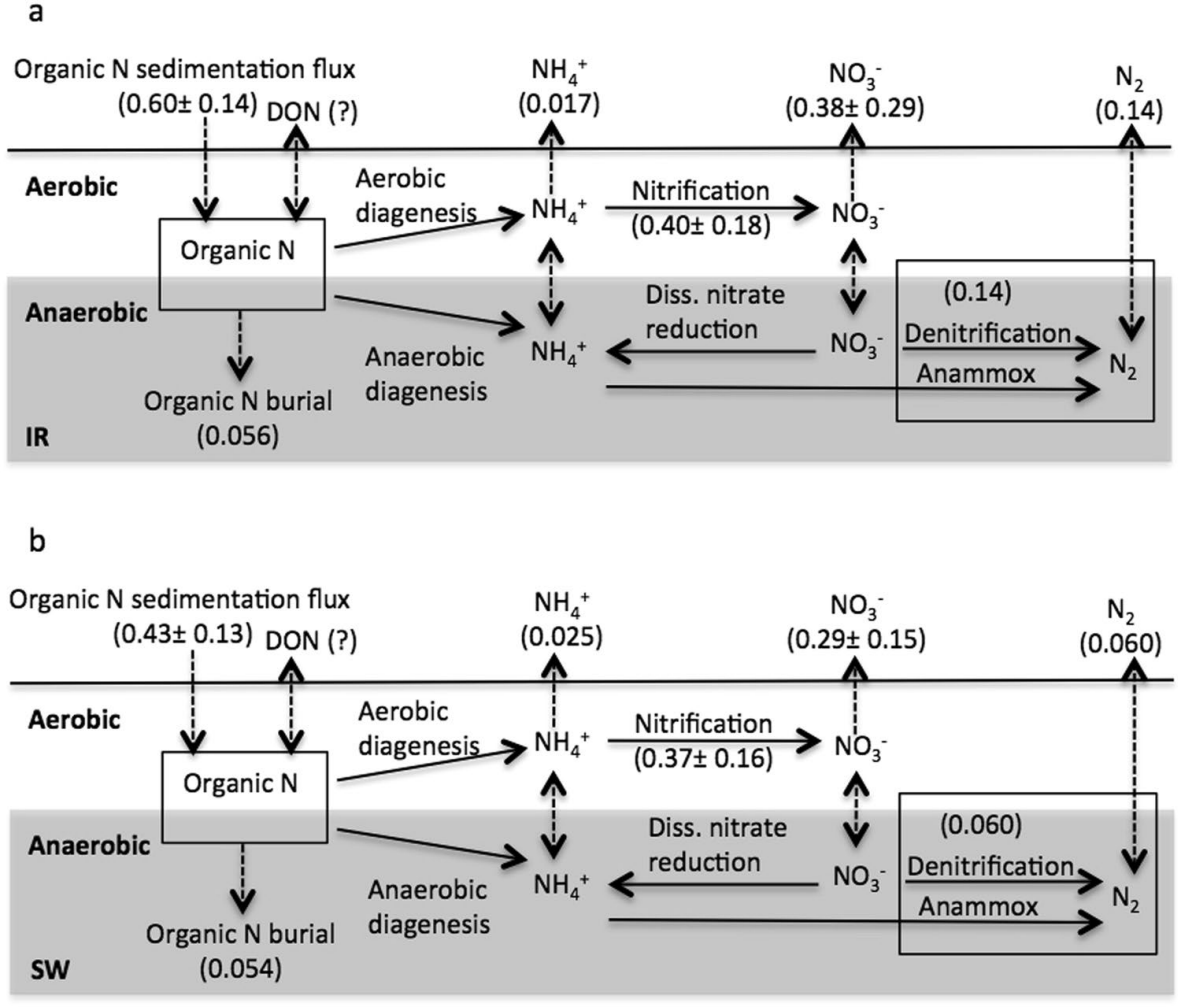
Mass balance for nitrogen in stations IR (**a**) and SW (**b**). Shaded area represents anoxic sediments. Descriptions of calculations are included in the materials and methods (mmol m^−2^ d^−1^).

To gain a quantitative appreciation for how anammox contributes to Lake Superior’s N-cycle, we have calculated as mass-balance on N for both stations (Fig. 3). N is largely supplied to the sediment through organic matter deposition. Only a small fraction of this N is ultimately buried, however, and much of the N deposited is returned to the water column either as NO_3_^−^ or as N_2_. Mass balance calculations yield N_2_ production rates that are very similar to those measured in our whole-core incubations giving us confidence that we have accurately captured the *in situ* rates and pathways. Anammox therefore plays an important role in overall N_2_ production, quantitatively influencing the amount of fixed N buried, the amount recycled to the water column, and thus overall N cycling in Lake Superior.

Oxygen and carbon cycling in Lake Superior compares well with trends observed in coastal marine environments^13^. The relative contribution of anammox to total N_2_ production also agrees with relative contributions determined in coastal marine sediments with similar water depths, maximum nitrate concentrations, and oxygen penetration depths^18,20^. Rates of anammox measured in Lake Superior, however, are lower than those typically measured in coastal marine sediments^18^. We hypothesize that this is related to the low rates of ammonium supplied through carbon degradation in Lake Superior sediments^13^.

Our experiments document appreciable anammox activity in Lake Superior’s sediments, showing that anammox can play an important role in the sedimentary N-cycle of freshwater environments. Our data also indicates that there are differences in the diversity of anammox bacteria between sites in Lake Superior, with a novel group of anammox bacteria likely present at the IR station. We find that the importance of anammox to total N_2_ production scales inversely with overall sediment biogeochemical activity in a fashion similar to that observed in marine sediments. We predict then, that anammox is most important in low productivity freshwater environments such as high-latitude lakes. As such, anammox would have global importance to continental N-cycling, and may be a key regulator of the productivity of inland ecosystems. This possibility should be tested through further studies to expand the geographical range of our observations and to more completely map the diversity of anammox bacteria on the continents.

### Material and Methods

#### Sample collection

Sediment samples were collected from stations SW and IR using the R/V Blue Heron. Sediment cores of 94-mm inner diameter were recovered using an Ocean Instruments multi-corer. The landing sites were monitored using a Knudsen 320/R echo sounder with a 28-kHz transducer to select flat areas with laterally homogeneous sediment accumulation.

#### Process rate measurements

Whole-core measurements of N-transformation rates were measured as previously described^17^ and only key differences in methodology are noted here. ^15^N-NO_3_^−^ was added to both the overlying water and injected into sideports in core tubes at 1 cm intervals to depths of 7 cm at SW and 5 cm at IR and to nominal final concentrations of 25 µM ^15^N-NO_3_^−^. The ratio of ^15^N/^14^N in the nitrate pool within the zone of nitrate reduction was estimated by measuring the isotopic composition of N_2_O, as previously described^17^. A total of 5 undisturbed intact sediment cores were incubated from each site and incubations terminated after 6, 16, and 38 hours. No relationship to incubation time was observed. The cores were incubated at 4 °C, which corresponds to the temperature (3–5 °C) of Lake Superior bottom waters from May to November. The cores were maintained at 4 °C in the dark and the overlying water was stirred at 60 revolutions min^−1^ using a magnetic stir bar suspended 3–4 cm above the sediment–water interface. Core tubes were sealed with thick rubber stoppers. Oxygen concentrations decreased over the course of the incubations, but not more than 10% from their *in situ* values and the measured decrease was linear throughout the incubation period.

Sediment slurries were prepared by mixing sediment from the top 0–3 and 3–6 cm of the core with an equivalent volume of bottom water that was previously purged with ultra-high-purity He gas to remove O_2_ and N_2_. The sediment slurry was subsequently incubated under He for an additional 24 h to remove residual N_2_ gas. Following this 24-h period, the sediment slurry was transferred, with no headspace, into ~12-ml gas tight vials (Exetainers, LabCo). Isotopic labels, and specific inhibitor (ATU, final concentration of 165 µmol L^−1^) were added. The sediment slurries were incubated at room temperature ~20 °C and mixed ~every 6 hours by inversion 3x. Upon sacrificing, 1 ml of slurry was removed from the Exetainer using a needle and syringe and replaced with He gas and 200 μl of a 37% formaldehyde solution to stop microbial activity. The withdrawn sediment slurry (1 ml) was filtered directly through a 0.2 μm pore size syringe filter and the filtrate was frozen for later analysis of NO_x_ and NH_4_^+^ concentrations. The formaldehyde-fixed sediment slurry was stored upside down in the Exetainers until isotopic analysis.

The isotopic composition of N_2_ was determined by injecting 25–50 μl of headspace gas into an in-house built injection system. Following injection, CO_2_ was trapped using Ascarite, N_2_ and N_2_O separated using a Poropak R GC column, and the sample stream passed through a reduction reactor to reduce N_2_O to N_2_ and O_2_ to H_2_O. H_2_O was trapped on Mg perchlorate, and the sample stream was introduced using a Conflo III to a Thermo Electron DELTA V plus IR-MS operated in continuous-flow mode. N_2_ was measured at masses 28, 29, and 30. Similarly, the N isotopic composition of N_2_O was measured by injecting 200–1000 μl of headspace gas, but the reduction reactor was bypassed and isotopic measurements were made on masses 44, 45, and 46. Measurements of ^15^N-NH_4_^+^ were conducted by converting NH_4_^+^ to N_2_ following oxidation by hypobromite. In the case of the slurry incubations, NH_4_^+^ was extracted in a 2 mol l^−1^ KCl solution prior to hypobromite oxidation and isotopic analysis.

#### Mass balance calculations

The N sedimentation flux at the sediment water interface (SWI) and in the deep sediments (burial) was calculated from carbon flux (12C:1N for Lake Superior sediments)^13^. The C burial flux was calculated from organic carbon concentrations and sedimentation rates measured by ^210^Pb. The carbon sedimentation flux (at the SWI) was carbon burial plus carbon remineralization. The latter was calculated from total oxygen uptake minus the oxygen consumed by nitrification. Fluxes of nitrate and ammonium are diffusive fluxes calculated using Fick’s Law, from the porewater concentration gradients. Nitrification rates were calculated by integrating nitrification rates in the surface sediments. Rates are second derivatives of the NO_x_ profile, and the integration is essentially the difference between nitrate flux at SWI and at the depth where nitrification goes to zero, i.e. the depth of oxygen penetration. This calculation though, is based on the assumption that zones of nitrification and nitrate reduction do not overlap^16^. Denitrification/Anammox rates were calculated by a mass balance approach. R(N_2_ removal) = Ammonium production by C remineralization − Flux out of ammonium + Flux out of nitrate. Ammonium production can be calculated from the rates of carbon mineralization and a 12C:1N ratio.

#### Molecular microbiology

Metagenomic DNA was extracted using the PowerLyzer™ PowerSoil® DNA Isolation Kit from MoBio following the manufacturers instructions. DNA was quantified spectrophotometrically (NanoDrop) and furthermore reviewed on a 1% agarose gel.

In order to analyze the hzsA genes present in our samples we conducted a nested PCR and prepared a clone library with the resulting products. In the first PCR round the *hzs*A gene was amplified with *hzs*A_526F (5′-TAYTTTGAAGGDGACTGG-3′) as forward and *hzs*A_1857R (5′-AAABGGYGAATCATARTGGC-3′) as reverse primer. The second round was performed with with *hzs*A_526F as forward and with *hzs*A_1829R (5′-TCATACCACCARTTGTA-3′) as reverse primer (for primer see Harhangi 2012). PCR reactions were performed in 50 µl end volume containing 5–10 ng template DNA respectively 1 µl of PCR product, 20 pmol of each primer, 1.25 Units *Taq* polymerase (Thermo), 40 nmol deoxynucleotides and 125 nmol MgCl_2_ in 1x *Taq* reaction buffer with KCl (Thermo). PCR reactions were performed as follows: 5 min at 96 °C followed by 35 resp. 32 cycles of 96 °C for 1 min, 53 °C resp. 51 °C for 1 min and 72 °C for 1.5 min. Final extension was performed at 72 °C for 5 min. Obtained PCR products were purified using the QIAEX® II Gel Extraction Kit from Qiagen and cloned into the pJET1.2/blunt cloning vector using the CloneJET PCR Cloning Kit from Thermo following the instructions of the manufacturers. Resulting clones were sequenced by Macrogen Europe (Netherlands) using the primers pJET1.2F (5′-CGA CTCAC TATAGGGAGA GCGGC-3′) and pJET2.1R (5′-AAGAACATCGATTTTCCATGG CAG-3′). In total 16 clones from each station were sequenced. In total 16 clones from each station were sequenced, of which 29 were *hzs*A genes without ambiguous bases. The sequences have been deposited in Genbank with the accession numbers MF565415 to MF565443.
